# Soluble IL-2 Receptor: A Biomarker for Assessing Myositis Activity

**DOI:** 10.1155/2014/472624

**Published:** 2014-02-04

**Authors:** Anne Tournadre, Jean-Jacques Dubost, Martin Soubrier, Marc Ruivard, Pierre Souteyrand, Jeannot Schmidt, Pierre Clavelou, Arlette Tridon, Jean-Michel Ristori

**Affiliations:** ^1^Department of Rheumatology, CHU Clermont-Ferrand, Hôpital Gabriel Montpied, 63003 Clermont-Ferrand, France; ^2^Department of Internal Medicine, CHU Clermont-Ferrand, Hôpital Estaing, 63003 Clermont-Ferrand, France; ^3^Department of Dermatology, CHU Clermont-Ferrand, Hôpital Estaing, 63003 Clermont-Ferrand, France; ^4^Emergency Department, CHU Clermont-Ferrand, Hôpital Gabriel Montpied, 63003 Clermont-Ferrand, France; ^5^Department of Neurology, CHU Clermont-Ferrand, Hôpital Gabriel Montpied, 63003 Clermont-Ferrand, France; ^6^Immunology Laboratory, Faculty of Pharmacy, 63003 Clermont-Ferrand, France

## Abstract

*Objective*. To evaluate the clinical significance of serum soluble IL-2R (sIL-2R) in inflammatory myopathies. *Methods.* Serum sIL-2R and CK levels were determined in 27 patients with IM during periods of disease exacerbation and inactive disease and were compared to 20 healthy controls and 23 controls with noninflammatory elevated CK. The performance of sIL-2R and CK tests for assessing disease activity was compared. *Results.* sIL-2R levels were increased in patients with IM. Significantly higher sIL-2R levels were detected in patients with disease exacerbation than in patients with inactive disease. In patients with IM, the sIL-2R levels correlated with the CK levels. Based on ROC analysis, diagnostic accuracy of sIL-2R and CK tests for disease activity was similar. However, when the CK threshold was defined by the upper limit of the normal, the specificity for the CK test dropped to 58%. *Conclusion.* Serum sIL-2R level could be useful to distinguish disease exacerbation from damage in IM, especially in patients with persistent elevated CK levels when a clinical muscular worsening is noted. For discrimination of the disease activity, CK testing requires the use of a different threshold than the upper limit of the normal.

## 1. Introduction

The idiopathic inflammatory myopathies (IM) are a group of chronic muscle diseases that share clinical symptoms characterized by muscle weakness and histological features with the presence in muscle tissue of inflammatory infiltrates. Based on clinical and histopathological features, they can be divided into polymyositis (PM), dermatomyositis (DM), and inclusion body myositis (IBM). In addition, new classifications distinguish immune-mediated necrotizing myopathy (IMNM), overlap myositis, and cancer-associated myositis [1, 2]. The evidence of a T-cell myocytotoxicity, the presence of autoantibodies, and the upregulation of MHC class I antigens in muscle cells suggest an autoimmune process [3]. DM is considered as a CD4-driven disease resulting in a microangiopathy affecting skin and muscle whereas PM is considered as a CD8-driven disease where muscle is the primary target of the immune attack. In DM, mononuclear cells consisting in perimysial CD4+ T cells and B cells predominate in perivascular area, MHC class I expression predominates on perifascicular fibers, and the reduction of capillary density contributes to perifascicular atrophy. In PM, endomysial CD8+ T cells and macrophages surround and invade nonnecrotic muscle fibers expressing MHC class I antigens. Activation of T cells can be assessed by measuring the serum level of the soluble form of the IL-2 receptor (sIL-2R). In IM, increased serum levels of sIL-2R compared to normal subjects have been reported [4–6] and serum sIL-2R levels varied with the disease activity [4, 7]. Muscle enzymes are widely used to assess activity and damage in IM. However, serum creatinine kinase (CK) or other muscle enzymes do not correlate well with disease activity [8, 9]. There would clearly be an advantage to have tools which assess disease activity and differentiate activity meaning features which have the capacity to be substantially improved by therapeutics from damage implying permanent change. In the present multicentric retrospective study, the clinical significance of serum sIL-2R measurement in relation to laboratory and clinical measures of disease activity was evaluated in patients with IM during period of disease exacerbation and/or inactive stages of the disease and compared to controls including patients with noninflammatory elevated CK levels.

## 2. Patients and Methods

### 2.1. Patients and Controls

Patient and control sera were analyzed for sIL-2R and CK levels. We used sera from 27 patients with IM (17 PM, 8 DM, and 2 IBM) and from 43 controls. Patients with PM and DM fulfilled the Bohan and Peter criteria for definite or probable diagnosis of PM and DM [10]. The diagnosis of IBM was established on the muscle biopsy showing mononuclear cell invasion of muscle fibers and vacuolated muscle fibers. The two patients with IBM incompletely fulfilled Griggs criteria [11], but they could be considered as probable IBM according the 2011 European Neuromuscular Centre diagnostic criteria for inclusion body myositis [12]. The clinical characteristics of the patients with IM are shown in Table 1. For control groups, we studied 20 normal subjects without evidence or history of muscular or autoimmune disease. Nine of them were investigated in cardiology department for chest pain or unexplained syncopes, 7 had a fibromyalgia, 3 had chronic back pain, and 1 had knee osteoarthritis. In addition, data from 23 patients with noninflammatory elevated CK levels were analyzed. Eleven patients had acute elevated CK (fall *n* = 3, fall with cerebral hematoma *n* = 3, seizure *n* = 4, and muscular trauma *n* = 1). Twelve others patients had persistent unexplained elevated CK without any muscle weakness. Six of them had persistent or transient myalgia, 4 were asymptomatic, and 2 had a fibromyalgia. Muscle biopsy was performed in 6 cases and was normal and electromyography performed in 8 patients. The study was approved by the local Research Ethics Committee of Gabriel Montpied Hospital and all subjects gave their informed consent.

### 2.2. Assessment of Disease Activity

Patients were considered with disease exacerbation if the treating physician strengthened the patient's immunotherapy.

### 2.3. Laboratory Analyses

From July 1991 to June 2003, blood samples were obtained paired with routine analyses. The sera were stored at −80°C until assayed in February and July 2003. sIL-2R concentrations were determined using a commercially available enzyme-linked immunosorbent assay according to the manufacturer's instructions (Beckman Coulter Inc., IM0559). The normal value indicated by the manufacturer was 70 picomoles (pM) ±45. CK levels were measured using standard methods in the hospital's chemistry laboratory (CK normal values: 10–147 UI/L). Commercialized dot-blots (Cyto-Dot, BMD, Antwerpen, Belgium, and Dot Bioadvance, Bussy Saint Martin, France) were used for Jo1 antibody identification.

### 2.4. Statistical Analysis

All group data were expressed as the mean value ± S.D. The Mann-Whitney *U* test was used for comparisons between two variables. Spearman's and Pearson's correlation coefficients were used to correlate any two variables with nonnormal and normal distribution, respectively. *P* values less than 0.05 were considered statistically significant. In order to evaluate the performance of serum sIL-2R and CK for the diagnosis of disease exacerbation, the sensitivity, specificity, and area under curve (AUC) were calculated using receiver operating characteristic (ROC) analysis. The analysis and the graphs were performed using GraphPad Prism 5 (version 5.02; GraphPad Software, La Jolla, CA, USA).

## 3. Results

### 3.1. Increased sIL-2R Levels in Patients with Inflammatory Myopathies

**Figure 1:**
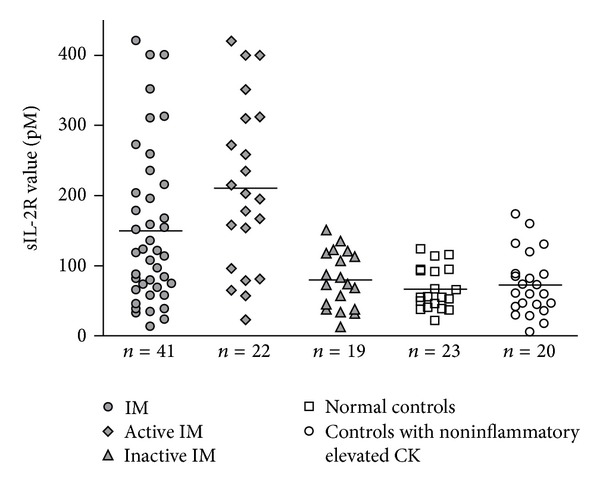
Serum sIL-2R values from 27 patients with IM, 20 normal control subjects, and 23 controls with noninflammatory elevated CK. Each data point represents the sIL-2R value and horizontal bars represent the mean for each group.

From the 27 patients with IM, 41 sIL-2R values (PM = 24, DM = 15, and IBM = 2) were analyzed and compared to the 43 controls (Figure 1). Each sIL-2R value corresponded to the mean of the sIL-2R assays determined during the same period of disease exacerbation or inactive disease as defined previously. In IM patients, 22 values were obtained during disease exacerbation and 19 during the inactive phase of the disease. 23 sIL-2R values were also obtained from healthy controls and 20 from controls with noninflammatory elevated CK. The sIL-2R serum levels were significantly higher in IM as compared to healthy controls and to controls with elevated CK (149.7 pM ± 112 versus 66.7 pM ± 30.15 and 72.4 ± 44.6, resp., *P* = 0.002 and *P* = 0.005). Significantly higher sIL-2R levels were observed in patients with disease exacerbation compared to patients with inactive disease (210.4 pM ± 119.2 versus 79.5 pM ± 40.3, *P* = 0.0002). There was no significant difference between PM and DM (145.3 pM ± 113 versus 154.4 pM ± 115.4, *P* = 0.9). Patients with IM or controls with noninflammatory elevated CK did not differ for the CK levels (1238.4 UI/L ± 2514.6 versus 676.5 UI/L ± 582.2, *P* = 0.4).

### 3.2. Correlation between sIL-2R and CK Levels in Patients with Inflammatory Myopathies

**Figure 2:**
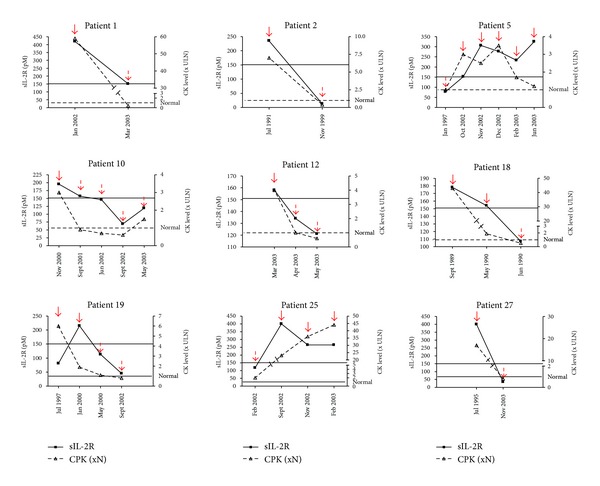
Correlation between serial measurements of sIL-2R and CK levels (times the upper limit of normal (x ULN)) for 9 patients at different time points. Red full arrows represent active disease and red dotted arrows inactive disease. For sIL-2R, the black line shows the cutoff value of 151 pM. For CK level, dotted line shows the normal value.

We next analyzed the relationship between the serum sIL-2R values, the CK levels, and the disease activity. In patients with IM, the sIL-2R levels correlated with the CK levels (*r* = 0.55, *P* = 0.0002). No significant correlation between sIL-2R and CK levels was observed for patients with disease exacerbation (*r* = 0.29, *P* = 0.19) or for patients with inactive disease (*r* = 0.20, *P* = 0.40). However, considering multivariate linear regression models with sIL-2R levels as dependent variable (after normalization using log transformation) and active and/or inactive disease and CK levels as covariates, the relationship between sIL-2R and CK levels was relevant (*P* = 0.05) in all patient analysis (active and inactive disease). In such model, including as covariate patients with active disease only, the relationship between sIL-2R and CK levels was not significant (*P* = 0.07) but was stronger (*P* < 0.05) compared to model with patients with only inactive disease (*P* = 0.31) suggesting a relationship between sIL-2R and CK levels in particular in patients with active disease, only statistically significant on all patient analysis because of a lack of power and a bimodal distribution depending on active and/or inactive disease. Changes in sIL-2R, CK levels, and disease activity were analyzed in 9 patients with IM (Figure 2). 5 PM, 3 DM, and 1 IBM had 2–6 serum samples collected at different time points in their disease course. Except in one patient (patient 19), sIL-2R values increased above the cutoff value of 151 pM during the disease exacerbation and were less than or equal to the cutoff value during the remission. In one patient (patient 25), CK level remained elevated during inactive disease. In 7 patients CK level changes were in accordance with sIL-2R values.

### 3.3. Diagnostic Performance of sIL-2R for Active IM

**Figure 3:**
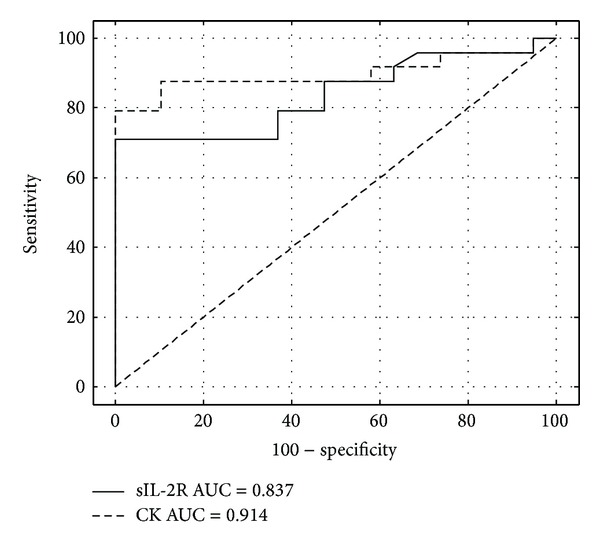
ROC plots of serum sIL-2R and CK values for identifying active myositis. ROC curves showed the best decision threshold in the upper left corner. The dotted line indicated the line of non-discrimination. AUC: area under curve.

**Figure 4:**
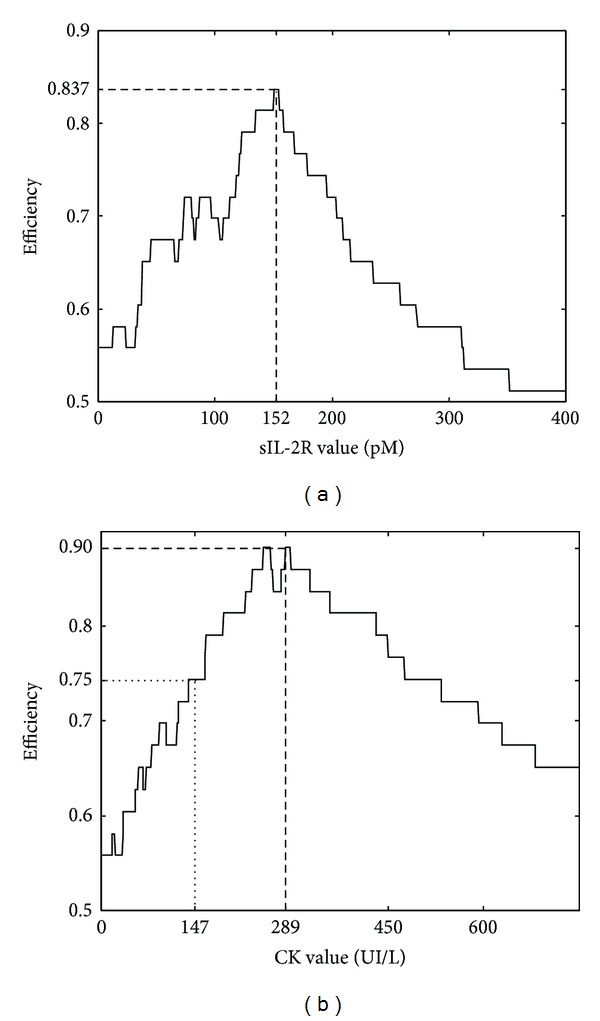
Test efficiency plots of serum sIL-2R and CK values for identifying active myositis. (a) Values of test efficiency peaked at a cutoff point of sIL-2R at 151 pM (dashed line). (b) 90% efficiency was obtained at the optimal cutoff value of CK at 289 UI/L (dashed line) but efficiency was 75% at the upper limit of the normal value of CK at 147 UI/L (dotted line).

We next compared the value of serum sIL-2R and the CK levels for assessing disease activity in the 27 patients with IM by a ROC analysis (Figure 3). The area under the curve (AUC) was similar for the sIL-2R and CK tests (0.837 with 95% confidence interval 0.783–0.978 and 0.914 with 95% confidence interval 0.689–0.933, resp., *P* = 0.3). For the sIL-2R test, the best cutoff decision point for the discrimination between disease exacerbation (positive results) and inactive myositis (negative results) was determined using the ROC analysis and the test efficiency (Figure 4). The best discrimination was achieved for a cutoff value of 151 pM with a test efficiency of 83.7%. The corresponding specificity and sensitivity were, respectively, 100% and 72% (Figure 4(a)). For the CK test, the best cutoff value for the diagnosis of disease exacerbation was 289 UI/L, with a test efficiency of 90%, a specificity of 100%, and a sensitivity of 82% (Figure 4(b)). However this optimal threshold was about twice the upper limit of the laboratory normal (147 UI/L). For the standard threshold, the test efficiency dropped dramatically to 76% with a specificity of 58% and a sensitivity of 91%.

## 4. Discussion

Consistent with previous studies [4–7], the serum sIL-2R levels were increased in IM patients compared to controls. Moreover, sIL-2R varied with the disease activity and significantly increased levels were found during the active phase of IM. As there was no significant increase in controls with noninflammatory elevated CK, we can suppose that the determinations of sIL-2R values were not affected by the levels of CK or by noninflammatory muscle damages. The presence in inflamed muscle of activated DCs and macrophages with IL-12 and IL-23 cytokines involved in the differentiation of Th1 and Th17 cells and the detection of both Th1 and Th17 cells in lymphocytic infiltrates of PM and DM muscle tissues strongly suggest an ongoing immune process involving activated T cells and DCs [13, 14]. Therefore, the serum sIL-2R could be, at least in part, released from activated T cells present in inflammatory infiltrates during the active phase of the disease and may serve as a useful tool to assess disease activity. In IM, distinguishing active inflammation from damages such as fibrosis, atrophy, or steroid-myopathy still remains a challenge but is crucial for appropriate treatment. Manual muscle strength testing is of limited value for differentiating the disease activity from damage. Because muscle biopsy is an invasive procedure, it can not be a serial method of assessing the myositis activity. Advances in imaging, particularly magnetic resonance imaging (MRI), have improved the assessment of the disease activity of patients with IM [15]. However, MRI can be normal even when there is an ongoing inflammation documented by muscle biopsy [16]. The level of serum CK, widely used to assess myositis activity, does not correlate well with muscle strength, muscle histopathology, or MRI in IM [8, 17]. Thereby, we determined the diagnosis performance of sIL-2R test for disease exacerbation and compared it with CK assay using ROC curve analysis. sIL-2R had a good diagnostic accuracy with an AUC of 0.837 but was not significantly different from the CK test. The specificity of the sIL-2R value at the decision threshold of 151 pM for the diagnosis of active disease was 100% whereas the specificity of the CK assay at the upper limit of the laboratory normal was only 58% indicating that, if the CK assay alone was a good indicator in case of disease exacerbation with a sensitivity of 91%, it was a relatively poor indicator in case of inactive disease as it can persist elevated. However, the specificity of the CK test for the diagnosis of disease exacerbation could be strongly improved when the threshold was increased approximately twice the upper limit of the normal. Therefore, even the diagnostic accuracy of CK test assessed using ROC plots suggested a good correlation with the illness activity; the discrimination of the disease activity based on a CK testing required the use of a different threshold. Keeping this in mind, the advantage of testing the sIL-2R level was not demonstrated by our measurements. However the sIL-2R measurement revealed a crucial advantage to establish the activity of IM when CK levels are persistently elevated. Indeed, elevated CK values are measured for various muscle disorders. In our study, sIL-2R levels were not significantly increased in controls with noninflammatory elevated CK. These findings indicate that the serial measurement of the serum sIL-2R level could be useful to distinguish the inflammatory activity from damage in patient with persistent elevated CK levels when a clinical muscular worsening is noted. There are, however, some limitations to our study. Also, this work is a retrospective study limited by the sample size of the patients who underwent serial measures during longitudinal followup and by the method used to assess the disease activity. The assessment of the disease activity and damage in IM still remains inadequate. No clinical, serologic, biochemical, imaging scans, or histological approaches are individually effective enough to be used as a gold-standard method in measuring the disease activity or damage. Therefore we selected a physician's global assessment of the disease activity corresponding to clinical practices. From a practical point of view, taken together with the previous observations [7], the determination of the serum sIL-2R levels together with clinical examination and MRI assessment might be useful for evaluation of disease activity in patients with residual muscle weakness and persistently abnormal CK levels despite treatment. An additional prospective study with longer observation period should be carried out to support these results.

## Figures and Tables

**Table 1:** Clinical data on patients with IM.

Patient	Age/sex	Diagnosis	Proximal muscle weakness	DM skin rash	↑ Muscle enzymes	Anti-Jo1 antibodies	Abnormal EMG findings	Abnormal muscle biopsy findings	Medication
1	20/F	PM/MCTD	+	−	+	−	−	+	Pred., MTX
2	46/F	PM	+	−	+	−	+	+	Pred., MTX
3	31/F	PM	+	−	+	−	+	+	Pred., MTX
4	65/F	PM	+	−	+	−	+	−	Pred., MTX
5	47/F	PM/ILD	+	−	+	+	NR	+	Pred., MTX, IVIG
6	72/H	PM	+	−	+	−	−	+	Pred.
7	67/F	PM	+	−	+	−	+	+	Pred.
8	27/F	PM/MCTD	+	−	+	−	+	−	Pred., AZA
9	41/H	PM	+	−	+	+	+	+	Pred., MTX
10	72/H	PM/HCV	+	−	+	−	+	+	Pred., IVIG
11	61/F	PM	+	−	+	−	+	+	Pred.
12	38/F	PM/ILD	+	−	+	+	+	+	Pred., MTX
13	61/H	PM	+	−	+	−	+	−	Pred., MTX, IVIG
14	57/F	PM/MCTD	+	−	+	−	+	+	Pred., MTX, chloroquine
15	77/F	PM	+	−	+	−	+	+	Pred.
16	63/H	PM/ILD	+	−	+	+	+	+	Pred.
17	48/F	PM	+	−	+	−	+	−	Pred., MTX, IVIG, AZA
18	37/H	DM	+	+	+	−	+	+	Pred., MTX
19	42/F	DM	+	+	+	−	+	−	Pred., MTX, IVIG
20	56/H	DM	+	+	+	−	+	−	Pred., MTX, chloroquine
21	44/H	DM	+	+	+	−	+	−	Pred., MTX, IVIG
22	14/F	DM	+	+	+	−	+	+	Pred.
23	67/H	DM	+	+	+	−	+	+	Pred.
24	40/F	DM	+	+	+	−	+	+	Pred., MTX
25	40/F	DM	+	+	+	+	+	+	Pred., MTX, IVIG, AZA, CSA
26	74/H	IBM	+	−	+	−	+	+	Pred.
27	69/F	IBM	+	−	+	−	+	+	Pred., MTX, IVIG

MCTD: mixed connective tissue disease; ILD: interstitial lung disease; HCV: hepatitis C virus; Pred.: prednisone; MTX: methotrexate; IVIG: intravenous immunoglobulin; AZA: azathioprine; CSA: cyclosporine; NR: not reported or not done.
